# To explore the effectiveness of atorvastatin in the postoperative formation of collateral blood vessels after encephaloduroarteriosynangiosis in patients with moyamoya disease: a prospective double-blind randomized controlled study

**DOI:** 10.3389/fneur.2023.1169253

**Published:** 2023-06-01

**Authors:** Gan Gao, Qian-Nan Wang, Fang-Bin Hao, Xiao-Peng Wang, Si-Meng Liu, Min-Jie Wang, Cong Han, Xiang-Yang Bao, Lian Duan

**Affiliations:** ^1^Chinese PLA Medical School, Beijing, China; ^2^Department of Neurosurgery, Chinese PLA General Hospital, Beijing, China

**Keywords:** moyamoya disease, atorvastatin, EDAS, collateral circulation, surgery

## Abstract

**Introduction:**

The aim of this large, prospective, double-blind randomized controlled trial is to investigate the effect of atorvastatin on the formation of collateral blood vessels in patients after encephaloduroarteriosynangiosis (EDAS) and to provide a theoretical basis for clinical drug intervention. Specifically, we will determine whether atorvastatin has an effect on the development of collateral vascularization and on cerebral blood perfusion after revasculoplasty in patients with moyamoya disease (MMD).

**Methods and analysis:**

Overall, 180 patients with moyamoya disease will be recruited and randomly assigned to the atorvastatin treatment group or the placebo control group in a 1:1 ratio. Before revascularization surgery, magnetic resonance imaging (MRI) scanning and digital subangiography (DSA) examination will be routinely performed on the enrolled patients. All patients will receive intervention via EDAS. According to the randomization results, patients in the experimental group will be treated with atorvastatin (20 mg/day, once a day, for 8 weeks) and patients in the control group will be treated with placebo (20 mg/day, once a day, for 8 weeks). All participants will return to the hospital for MRI scan and DSA examination 6 months after EDAS surgery. The primary outcome of this trial will be the difference in the formation of collateral blood vessels revealed by DSA examination at 6 months after EDAS surgery between the two groups. The secondary outcome will be an improvement in the dynamic susceptibility contrast sequence cerebral perfusion on MRI at 6 months after EDAS, compared to the preoperative baseline.

**Ethics and dissemination:**

This study was approved by the Ethics Committee of the First Medical Center of the PLA General Hospital. All participates will voluntary provide written informed consent before participating in the trial.

**Clinical trial registration:**

ClinicalTrials.gov, ChiCTR2200064976.

## 1. Introduction

Moyamoya disease (MMD) is a rare, chronic, and progressive cerebrovascular disorder characterized by stenosis and occlusion of the distal carotid, proximal middle, and anterior cerebral arteries and is accompanied by the development of small collateral vessel networks (1, 2). If the newly-formed collateral vessels do not maintain sufficient cerebral perfusion, it can lead to ischemic events, particularly in pediatric patients who require more blood flow for cerebral development and maturation.

At present, there is no effective drug for treating MMD, and revascularization surgery for symptomatic MMD is considered the standard treatment for preventing further strokes. Cerebrovascular reconstruction can form new collateral networks, improve cerebral blood flow reserve, and reduce the occurrence of secondary strokes, and has become the preferred approach of many neurosurgeons (3). Encephaloduroarteriosynangiosis (EDAS) is one of the most used indirect revascularization procedures and is a widely established treatment strategy for patients with MMD due to its excellent postoperative results, leading to extensive collateral vessels formation and minimal complications. However, in our previous long-term follow-up studies, we found that approximately 20% of patients had poor postoperative collateral circulation (4, 5). A recent study performed by our center showed that the formation of collateral vessels in EDAS is primarily driven by angiogenesis, and the endothelial progenitor cell (EPC) count may be the most critical factor for promoting the development of collateral networks (6). An increase in the proliferation and activation of EPCs may lead to better collateral circulation in patients.

Atorvastatin, a classic lipid-lowering drug, has been widely used in the cardiovascular field. Earlier literature indicated that statins can affect the mobilization, proliferation, chemotaxis, and apoptosis of EPCs (7, 8). Our previous retrospective study found that the extent of postoperative formation of collateral vessels in patients with atorvastatin treatment was significantly higher than in the control group (9). This provides a novel option in the treatment of MMD, which may ensure a better outcome in patients receiving EDAS surgery. However, the benefit remains to be confirmed by a large prospective randomized controlled study.

Therefore, we plan to explore whether atorvastatin can promote the formation of collateral vessels after EDAS and provide evidence for its beneficial use as adjuvant treatment in MMD.

## 2. Methods and analysis

### 2.1. Study design

**Figure 1:**
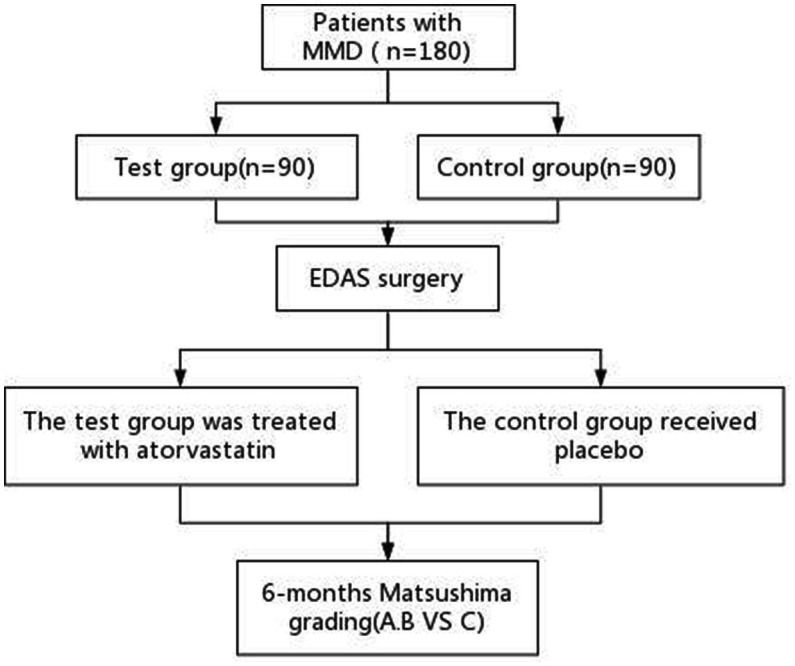
Study flow chart. MMD, moyamoya disease; EDAS, encephaloduroarteriosynangiosis.

This will be a single-center, prospective, randomized, double-blind, placebo-controlled study (Figure 1). A total of 180 patients will be recruited from the Department of Neurosurgery, Chinese PLA General Hospital, and randomly assigned to the atorvastatin treatment or the placebo control group at a ratio of 1:1. The study is planned to run from October 2022 through October 2025.

### 2.2. Inclusion and exclusion criteria

Participants will be recruited from the hospital wards. Inclusion criteria are as follows: (a) patients are diagnosed with MMD according to the 2021 Japanese Guidelines for the “Management of Moyamoya Disease: Guidelines from the Research Committee on Moyamoya Disease and Japan Stroke Society,” where DSA or magnetic resonance angiography shows stenosis or occlusion at the distal end of the internal carotid artery or anterior cerebral artery and/or middle cerebral artery, and abnormal proliferation and smoky vascular network can be found near occluded or stenosis vessels in the arterial phase of angiography (10); (b) patients are at a stable stage of the stroke, without acute or subacute cerebral infarction or cerebral hemorrhage, and more than 3 months after the last cerebral infarction or cerebral hemorrhage; (c) age ≥ 18 years; (d) no previous intracranial and extracranial vascular reconstruction; (e) complete understanding of the nature and content of the study, voluntary participation, and provision of written informed consent.

Exclusion criteria are as follows: (a) long-term statin therapy; (b) long-term steroid therapy; (c) acute or subacute cerebral infarction or cerebral hemorrhage; (d) allergy to statins or their ingredients; (e) abnormal renal and liver function; (f) uncontrolled hepatitis and other liver diseases, as well as other diseases that may interfere with the study; (g) pregnancy or lactation; (h) meningitis, autoimmune diseases, multiple neurofibromatosis, down syndrome, craniocerebral trauma, intracranial tumors, radiation damage, and other underlying diseases that may cause smog; (i) participation in a different experimental treatment study at the same time; and (j) failure to successfully complete the test due to poor compliance.

### 2.3. Randomization and treatment allocation

All enrolled patients will be randomly assigned to the atorvastatin treatment group or the placebo control group in a 1:1 ratio (*n* = 90 for each). The randomization sequence will be obtained according to the SPSS software program, and the randomization process will not be stratified. The randomized sequences of group assignments are then hidden in sequentially numbered opaque, closed envelopes. Atorvastatin (Pfizer) and placebo will be stored at the participating hospitals (20–30°C, while avoiding direct lighting), and they will be handled exclusively by the study nurses responsible for drug storage, distribution, and documentation. Placebo tablets are made from dextrin and have the same weight and appearance as atorvastatin. Two types of tablets are made by Shandong ARURA Pharmaceutical Research and Development Co., based on a randomized sequence, and are numbered sequentially. The randomization information and drug type are not publicly available to all investigators, competent physicians, and patients. All patients will be numbered sequentially, and the 8-week dose will be dispensed by the medication nurse in numbered order. At the end of the follow-up visit for the last enrolled patient, the sealed, opaque envelope will be opened by the principal investigator with unblinding (11).

### 2.4. Intervention

Patients in both groups will undergo standard EDAS procedures. EDAS surgery will be performed by experienced neurosurgeons. The main steps are as follows: (i) in accordance with the blood supply artery, contour the scalp with an incision design, (ii) cut the skin and subcutaneous free out long enough blood supply arteries, (iii) drill a hole on both ends of the bone flap, (iv) form an ellipse milling cutter governor of bone flap, (v) cut an open bar and epidural of about 1-cm width, (vi) maintain epidural blood vessels as far as possible, (vii) sew with fascia artery blood supply on cut epidural, and (viii) return the flap and fix it. The supplying artery remains open.

According to the results of randomization, patients in the experimental group will receive atorvastatin (20 mg/day, once a day, for 8 weeks), and patients in the control group will receive placebo (20 mg/day, once a day, for 8 weeks) from the first day after surgery (11). This dosage was chosen because it had minimal side effects in hyperlipidemia (12, 13), and because a high dose of atorvastatin (80 mg) was shown to increase the risk of bleeding in patients with low body weight and uncontrolled hypertension in a stroke prevention trial that focused on aggressively lowering cholesterol levels (14). Liver and kidney function tests will be performed every 4 weeks during treatment. All drug-related adverse reactions will be communicated to the investigators in a timely manner, and the investigators will decide whether to conduct clinical intervention or withdraw from the study according to the patient-specific situation. All drug-related adverse effects will be documented in detail.

### 2.5. Outcomes

#### 2.5.1. Safety outcomes assessment

We will evaluate the safety of atorvastatin calcium tablets based on the following events: (1) presence of abnormal liver and kidney function; (2) severe adverse drug reactions, such as death, life-threatening or permanent or severe disability, permanent organ dysfunction, emergency hospitalization or prolonged hospitalization; and (3) new strokes occurring during follow-up after EDAS.

#### 2.5.2. The primary outcomes

The main outcome will be the formation of collateral blood vessels in patients 6 months after EDAS and it will be measured by DSA. Additionally, we will compare whether there are statistical differences between the experimental and the control group and analyze whether there is a correlation between the effect of vascular reconstruction and the application of atorvastatin.

The standard evaluation of the formation of collateral vessels relies on the Matsushima grading: grade A, the superficial temporal artery compensates to the intracranial mass, covering more than 2/3 of the blood supply area of the middle cerebral artery; grade B, the superficial temporal artery compensates to the intracranial middle one in equal amount, covering 1/3–2/3 of the blood supply area of the middle cerebral artery; grade C, there is no communication between the superficial temporal artery and the intracranial one, no growth to the intracranial artery, or a small amount of compensation from the superficial temporal artery to the intracranial one, covering 0–1/3 of the blood supply area of the middle cerebral artery (15).

#### 2.5.3. The secondary outcomes

Dynamic Susceptibility Contrast-Magnetic Resonance Imaging examination will be performed to evaluate the improvement of cerebral hemodynamic parameters at 6 months after EDAS, compared to the preoperative baseline level. The time to peak contrast will be used to assess the patient’s hemodynamic status, with mean transit time and hemodynamic parameters calculated by perfusion (16).

The modified Rankin Scale (mRS) score will be used to evaluate the improvement of clinical symptoms of the patients before surgery and at 6 months after surgery. The mRS scores will be used to evaluate the degree of disability or dependence in daily activities of the patients, and the improvement of mRS score in the two groups will be compared and analyzed before surgery and 6 months after surgery (17).

## 3. Results

All participants’ data required for measurement will be collected and analyzed prior to unblinding. All outcome measures will be evaluated by two researchers, and consensus on the results will be required. If there is no consensus, another researcher will join the evaluation, and serve as arbitrator. Finally, the results will be collected by the principal investigator, and the group allocation information will be unblinded and statistically analyzed. The number of patients who experienced adverse events related to the atorvastatin calcium tablet intervention will be determined by the principal investigator.

### 3.1. Data monitoring agency

An independent data safety and monitoring committee consisting of clinicians and principal investigators will be established to oversee the overall conduct of the study. Regular meetings will be held to assess the occurrence of adverse events.

### 3.2. Sample size estimation

Using the normal approximation method and Pearson Chi-square test sample size calculation formula for the rate comparison between the two groups, test level *α* = 0.05 and test efficiency 1−β = 0.80 were set. According to the preliminary experimental results of this project, the probability of formation postoperative collateral networks (grade A or grade B) in patients with MMD treated with atorvastatin was 79.1%. The incidence in development of postoperative collateral vessels (grade A or B) in patients with MMD who were not treated with atorvastatin was 58.2%. Assuming that the coefficient of sample size relationship between the experimental group and the control group, K, is equal to 1, the minimum sample size for the two groups was calculated as *n* = 77, with the estimated loss to follow-up rate and rejection rate at 10%. The final total minimum sample was *n* = 154/0.9 = 172. We ultimately began with 180 cases.

### 3.3. Statistical analysis

All data will be analyzed by intention-to-treat and per-protocol. Categorical variables will be analyzed by chi-square test and continuous variables will be compared by independent *t*-tests or ANOVA. A U-test will be performed for variables that do not follow a normal distribution. Univariate analysis will be used to assess age, sex, initial symptoms at diagnosis, collateral grade, posterior circulation involvement, and use of atorvastatin. Variables significantly associated will be selected and analyzed by multiple linear regression. A value of *p* < 0.05 will be considered statistically significant. SPSS 26.0 software will be used for statistical analysis of all data.

## 4. Discussion

At present, the etiology and pathogenesis of MMD are unknown, and there are no effective therapeutic drugs. The main purpose of treatment in MMD is to improve cerebral blood flow, and extracranial and intracranial revascularization surgery is an effective mean to improve cerebral blood circulation and reduce the risk of secondary strokes (18). EDAS contributes to the formation of collateral vessels in patients with MMD. However, the mechanism of collateral vessel formation after EDAS is still unclear, which leads to a lack of interventions to promote the formation of collateral networks and accelerate the speed of blood flow reconstruction.

Earlier, we found that some patients with MMD have poor development of collateral vessels after EDAS. In our previous study, cerebral artery angiography was performed at 6 months after EDAS, and the Matashima grading of collateral vessel formation was applied to patients, among which 54.2–78.4% had good collateral circulation (grade A or B) and 21.6–45.8% had poor collateral circulation (grade C) (19). Therefore, there is an urgent need for safe and effective drugs to promote the formation of bridging arteries after EDAS and further improve the therapeutic effect of revascularization.

Compared with other statins, atorvastatin calcium tablets have the advantage of stronger regulation of dyslipidemia, shorter half-life, and higher bioavailability (20). Atorvastatin has been widely used in cardiovascular and cerebrovascular diseases as a lipid-lowering and plaque stabilizing drug. Additionally, atorvastatin can promote angiogenesis in stroke and brain injury models (21–23). However, to the best of our knowledge, there have been no reports on the ability of any drug, including atorvastatin, to promote the formation of collateral vessels after EDAS in patients with MMD.

First, since there is no previous study on the efficacy of statins in the treatment of MMD, the dose of atorvastatin used in this study is based on to earlier studies in other neuronal diseases, which means this may not be the optimal dose for patients with MMD. In addition, stratified randomized design will not be carried out in this study. The consistency of baseline characteristics and Suzuki stage of patients in the two groups can only be verified by concordance correlation coefficient, and there may be potential deviations in the results.

In conclusion, we expect to provide new therapeutic options for medically-assisted treatment of MMD by conducting a prospective double-blind randomized controlled study on the formation of collateral blood vessels after statin-induced vascular reconstruction surgery. These results will provide a valuable theoretical basis for larger multi-center clinical trials in the future.

## Ethics statement

The studies involving human participants were reviewed and approved by Chinese PLA general Hospital. The patients/participants provided their written informed consent to participate in this study.

## Author contributions

GG, X-YB, and LD conceived the study. GG and CH supervised and coordinated all aspects of the work. GG, Q-NW, F-BH, and LD designed the experiments. GG, Q-NW, and X-PW wrote the paper. GG, S-ML, and M-JW performed the experiments, analyzed the data, and prepared the figures and tables. F-BH and X-PW contributed with analytical tools. All authors contributed to the article and approved the submitted version.
